# Evaluation of buccal swabs for pharmacogenetics

**DOI:** 10.1186/s13104-018-3476-5

**Published:** 2018-06-14

**Authors:** J. Sidney Ang, Martin N. Aloise, Diana Dawes, Maryn G. Dempster, Robert Fraser, Andrea Paterson, Paul Stanley, Adriana Suarez-Gonzalez, Martin Dawes, Hagit Katzov-Eckert

**Affiliations:** 1 GenXys Health Care Systems Inc., Vancouver, BC Canada; 2 Department of Physical Therapy, University of British Columbia, Vancouver, BC Canada; 3 Shoppers Drug Mart Corporation, Vancouver, BC Canada; 4 Molecular You Inc., Vancouver, BC Canada; 5 Personalized Medicine Initiative, University of British Columbia, Vancouver, BC Canada; 6 Clinicare Pharmacists Inc, Vancouver, BC Canada; 7 Department of Family Practice, University of British Columbia, Vancouver, BC Canada

**Keywords:** Sample collection, Buccal swabs, OpenArray, Copy number variation, Pharmacogenetics

## Abstract

**Objective:**

A simple, non-invasive sample collection method is key for the integration of pharmacogenetics into clinical practice. The aim of this study was to gain samples for pharmacogenetic testing and evaluate the variation between dry-flocked and sponge-tipped buccal swabs in yield and quality of DNA isolated.

**Results:**

Thirty-one participants collected samples using dry-flocked swabs and sponge-tipped swabs. Samples were assessed for DNA yield, quality and genotyping performance on a qPCR OpenArray platform of 28 pharmacogenetic SNPs and a *CYP2D6* TaqMan copy number variant. DNA from sponge-tipped swabs had a significantly greater yield compared to DNA collected with dry-flocked swabs (p = 4.4 × 10^−7^). Moreover, highest genotyping call rates across all assays and highest CNV confidence scores were observed in DNA samples collected from sponge-tipped swabs (97% vs. 54% dry-flocked swabs; 0.99 vs. 0.88 dry-flocked swabs, respectively). Sample collection using sponge-tipped swabs provides a DNA source of sufficient quantity and quality for pharmacogenetic variant detection using qPCR.

**Electronic supplementary material:**

The online version of this article (10.1186/s13104-018-3476-5) contains supplementary material, which is available to authorized users.

## Introduction

Pharmacogenetics is a rapidly advancing field that has the potential to improve the safety and efficacy of medications by individually tailoring dosage and drug choices based on genetic profiles [1–3]. There is increasing evidence for the clinical utility of pharmacogenetics [2, 4]; guidelines from the Clinical Pharmacogenetics Implementation Consortium (CPIC) and the Dutch Pharmacogenetics Working Group (DPWG) that outline drug-gene interactions, are facilitating the clinical use of pharmacogenetic test results [5–7]. For clinicians to integrate pharmacogenetics into their care and use this knowledge, there needs to be an easy, cheap method of collecting samples that results in DNA of good quality.

Clinical diagnostics and genetic studies have traditionally used blood samples. Obtaining blood is invasive, costly, and may not be necessary for pharmacogenetics, where easy to collect, less costly alternatives such as saliva and buccal swabs can be used. In a published study, we used the Oragene saliva collection kit (DNA Genotek, Ottawa, ON); saliva samples resulted in a high mean DNA yield (6.0 ± 3.9 µg) [4]. Other studies have also shown high DNA yield from the Oragene saliva collection kit [8–10]. Although saliva sampling for genetic testing has gained acceptance [11–13], some patients in our study found it difficult to spit into a tube while others found it difficult to produce enough saliva (2 ml per sample). Dry mouth is a common side effect of medications and can hinder saliva collection. Hence, we sought to evaluate alternative sample collection methods for pharmacogenetic testing.

Evidence varies on the use of buccal cell DNA for genotyping [14], with a tendency for buccal swabs to yield less DNA compared to saliva collection [15–17]. More recent studies have evaluated the usefulness of swabs for genetic applications (see Additional file 1). To provide an easy way for patients to collect samples at the clinic, pharmacy or in the comfort of their own homes, we evaluated the performance of two swab types available on the market: a dry-flocked swab (Puritan PurFlock) and a sponge-tipped swab with a stabilizing solution (Oragene ORAcollect). We compared the DNA yield and genotyping quality from the swabs on a pharmacogenetic panel of 28 single nucleotide polymorphisms (SNP) and a cytochrome P450 family 2 subfamily D member 6 (*CYP2D6)* copy number variation (CNV) assay.

## Main text

### Materials and methods

#### Sample collection

Participants aged 18 years and over were invited to join the study via email notifications and advertisements placed on bulletin boards at the University of British Columbia, Vancouver BC. Participants were asked to provide samples using dry-flocked buccal swabs (Puritan PurFlock, Fisher Scientific, Waltham MA) and sponge-tipped buccal swab (Oragene ORAcollect, DNA Genotek, Ottawa, ON) using self-guided instructions; the order in which these samples were given was computer randomized. A research assistant was present during sample collection to answer questions participants had regarding the instructions for sample collection. All samples were stored at room temperature. Pharmacogenetic reports were not given to participants, as this was not within the scope of this study.

#### DNA extraction

DNA was extracted using the Ambion MagMAX bead-based DNA extraction kit (Applied Biosystems, Foster City, CA) according to the manufacturer’s instructions. DNA was extracted 3 days after sample collection and eluted in 60 µl. DNA quantity and quality were assessed using Qubit 2.0 fluorescence assay (ThermoFisher Scientific, Waltham MA). The purity of samples was determined by the optical density A260/280 (OD_A260/280_) values using NanoVue spectrophotometer.

#### Genotyping

Genotyping was performed on the QuantStudio 12K Flex quantitative polymerase chain reaction (qPCR) instrument (LifeTechnologies, Carlsbad, CA) on assays validated by the manufacturer (ThermoFisher Scientific, Waltham MA). The content of the assays included 28 SNPs influencing drug response on the OpenArray platform (see Additional file 2) and a *CYP2D6* CNV TaqMan assay (Assay ID: Hs_00010001_cn). To validate assays, 44 DNA controls from the National Institute of General Medical Sciences (NIGMS), Human Genetic Cell Repository at the Coriell Institute for Medical Research were genotyped. Concordance of calls was confirmed with the expected genotype. In addition Sanger sequencing of the control DNA verified results. For each run, participant samples were run in duplicates on the OpenArray and quadruplicates on the CNV assay along with Corriell DNA samples used as positive controls and no template controls (NTCs).

#### Statistical analysis

The TaqMan Genotyper software v1.3.1 (Applied Biosystems) was used to determine SNP genotyping call rates on the OpenArray. Genotype call rate was calculated for each OpenArray assay, dividing the total number of successfully assigned genotypes by the total number of attempted genotype assignments.

For the CNV TaqMan assay CopyCaller v2.1 software (Applied Biosystems, Foster City, CA) was used to assign *CYP2D6* copy number calls and CNV confidence scores. DNA samples were assigned a wild type diploid copy number and copy number variation (deletion or duplication events) were grouped in the 2N and CNV groups, respectively. A 95% genotyping call rate and 95% confidence score was set for SNP and CNV genotyping results, respectively. A two-sample Student’s t-test assuming unequal variances was calculated to determine the significant difference between DNA yield, purity and call rates from the different DNA types in pairs. The genotyping assignments generated from the DNA sample type (sponge-tipped vs dry-flocked) from the same participant were compared to calculate concordance.

### Results

Thirty-one participants were recruited and collected sponge-tipped swabs and dry-flocked swabs. Participants found self-guided instructions for collection straightforward and easy to follow for both types of swab. Participants raised questions on the necessary pressure for rubbing the swabs and the correct placement of swabs in the mouth. Comments from participants about the two swabs were similar with some preferring sponge-tipped swabs and others preferring the dry-flocked swabs; there was no consensus of opinion.

There was a significant difference in mean DNA yield between DNA collected using dry-flocked swabs versus sponge-tipped swabs (0.26 ± 0.34 µg vs. 1.91 ± 1.44 µg p = 4.4 × 10^−7^; Table 1). Mean DNA purity across all collection types were in the accepted range of for pure DNA (mean (SD)) 1.72(0.78) and 1.85(0.39) for dry swabs and sponge-tipped swabs, respectively.

**Table 1:** Quantity and purity of DNA from sponge-tipped and dry-flocked swabs

Swab type	Yield (µg)				Purity (OD_A260/A280_)			
	Mean	SD	Min	Max	Mean	SD	Min	Max
Sponge-tipped swabs (n = 31)	1.91	1.44	0.26	5.57	1.85	0.39	0.65	2.43
Dry-flocked swabs (n = 31)	0.26	0.34	0.06	1.46	1.72	0.78	0.14	3.10
Significance	p-value = 4.4^−7^				p-value = 0.4			

**Fig. 1:**
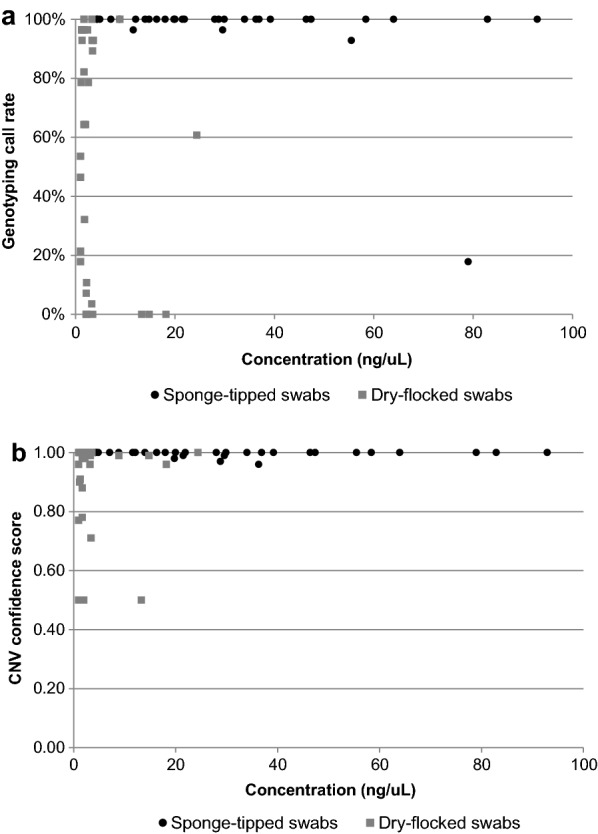
Comparisons of concentration on assay performance **a** OpenArray of 28 SNPs, **b** *CYP2D6* CNV assay

There were three samples collected from sponge-tipped swabs that had low DNA concentrations (< 5 ng/µl). These three samples had 100% genotyping call rates on the OpenArray and 100% CNV confidence scores (Fig. 1). Conversely, one of the samples that had DNA concentration of 79 ng/µl had the lowest OpenArray call rate (18%). The highest call rates on the OpenArray (> 95%) for dry-flocked swabs were observed for seven samples with concentration ranging between 1.2 and 8.9 ng/µl (Fig. 1a). The highest CNV confidence calls (> 95%) were observed for samples with concentration ranging between 1.0 and 24.4 ng/µl (Fig. 1b).

**Fig. 2:**
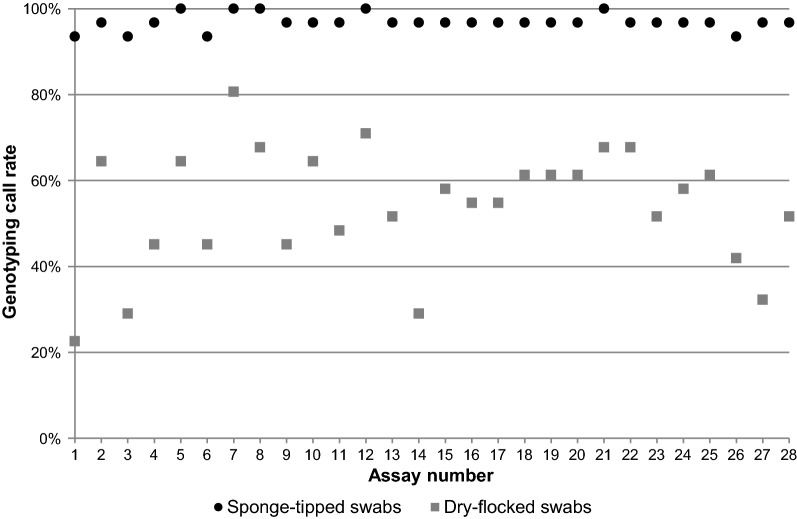
Mean genotyping call rate across a 28 SNP pharmacogenetic panel on the OpenArray platform

For all the assays on the OpenArray, DNA samples collected from sponge-tipped swabs resulted in overall higher genotyping call rates (97%) compared to dry-flocked swabs (54%) (Fig. 2). With an added step of a 0.5 × dilution of extracted DNA, the sponge-tipped swabs had consistent genotyping call rate > 95%. Sample call rate on the OpenArray varied by individual with 29(93.5%) participants having sample call rates > 95% from sponge-tipped swab DNA and 4(12.9%) having sample call rates > 95% from dry-flocked swab DNA. A 94% concordance of successfully genotyped assays was observed between sponge-tipped swabs and dry-flocked swabs collected from the same individual.

Copy number calls for *CYP2D6* were assigned to 2(6.5%) of the samples from sponge-tipped swabs with an overall confidence score of 0.99. In comparison, 8(25.8%) of the samples from dry-flocked swabs were assigned copy number calls with an overall confidence score of 0.91 (see Additional file 3).

### Discussion

The purpose for this research is to help investigators, and companies, identify an optimal collection method that yields high-quality DNA for genotyping. This study provides new information on the DNA quality for two types of swabs, which was high (defined as OD_A260/A280_ 1.8) [18, 19] though there was a significant difference in DNA yield (p-value = 4.4^−7^). On the OpenArray platform, sponge-tipped swabs had the highest overall genotyping call rate (97% vs. 54%) and highest confidence score for *CYP2D6* CNV TaqMan Assay (0.99 vs. 0.91). A > 95% SNP genotyping call rate is considered to reflect acceptable quality data and CNV confidence score of 99% is considered high quality [20–22].

One DNA sample isolated from the sponge-tipped swab had a call rate below 95%. In routine lab practice, samples with low call rates would be recollected due to incomplete results for patient reports. Omitting this sample increased the overall genotyping call rate to 99.5% for the sponge-tipped swabs.

*CYP2D6* CNV results showed an increase of CNV assignments for DNA collected from dry-flocked swabs 8(25.8%) compared to sponge-tipped swabs 2(6.5%). Higher number of CNV assignments correlated with lower confidence scores. The majority of participants 30(97%) were able to collect samples that resulted in high genotyping call rates for the sponge-tipped swab while only a few individuals 4(13%) were able to collect high call rate samples for both types of swabs. In conclusion, sponge-tipped swabs were acceptable to patients, and provided good quality DNA of sufficient yield for qPCR based pharmacogenetic testing.

## Limitations

The study provides support for an easy to collect swab for pharmacogenetic testing. Limitations to the study include small sample size and possible differences in sample collection technique between individuals. Moreover, only two types of swabs from those available on the market were tested, and the study did not include comparisons with other types of collection methods such as blood or saliva from the same individual.

## Additional files

**Additional file 1.** Summary of studies evaluating collection types for various genetic applications.

**Additional file 2.** List of SNPs on the OpenArray panel.

**Additional file 3.** Copy number calls for DNA samples genotyped on a *CYP2D6* CNV TaqMan Assay.

## Abbreviations

CPIC: Clinical Pharmacogenetics Implementation Consortium
DPWG: Dutch Pharmacogenetics Working Group
SNP: single nucleotide polymorphism
CYP2D6: cytochrome P450 family 2 subfamily D member 6
OD_A260/280_: optical density A260/280
CNV: copy number variation
qPCR: quantitative polymerase chain reaction
NIGMS: National Institute of General Medical Sciences
NTC: no template control
