# Supplementary material for: Evaluation of buccal swabs for pharmacogenetics
Source: BMC Res Notes. 2018 Jun 14;11:382. doi: 10.1186/s13104-018-3476-5 (PMC6000964; doi:10.1186/s13104-018-3476-5)
Supplement: Supplementary file 1 — Additional file 1. Summary of studies evaluating collection types for various genetic applications. [file 13104_2018_3476_MOESM1_ESM.docx]

**Additional file 1** Summary of studies evaluating collection types for various genetic applications

| **Year** | **Collection methods** | **Study size n** | **Conclusion** | **Collection device** | **Reference** |
| --- | --- | --- | --- | --- | --- |
| 2017 | Saliva, dry swab, blood | 7 | Differences in qPCR CNV genotyping calls from samples collected from the same individual dependent on DNA source | Catch-all swab | [[1](#_ENREF_1)] |
| 2016 | Dry swab | 10, 063 | HLA typing using next generation sequencing with swabs achievable | LabCorp cotton tip swab | [[2](#_ENREF_2)] |
| 2015 | Saliva tube, Dry swab | 104 | Buccal swab are more stable over time for PCR applications | not specified | [[3](#_ENREF_3)] |
| 2012 | Dry swab, blood | 16 | Swabs result in lower yield and lower genotyping quality | not specified | [[4](#_ENREF_4)] |
| 2011 | Saliva tube, Dry swab | 14, 019 | Saliva samples had higher yield and high quality for TaqMan assays | Oragene | [[5](#_ENREF_5)] |

**References**

1. Ooi DS, Tan VM, Ong SG, Chan YH, Heng CK, Lee YS: **Differences in AMY1 Gene Copy Numbers Derived from Blood, Buccal Cells and Saliva Using Quantitative and Droplet Digital PCR Methods: Flagging the Pitfall.** *PLoS One* 2017, **12:**e0170767.

2. Yin Y, Lan JH, Nguyen D, Valenzuela N, Takemura P, Bolon YT, Springer B, Saito K, Zheng Y, Hague T, et al: **Application of High-Throughput Next-Generation Sequencing for HLA Typing on Buccal Extracted DNA: Results from over 10,000 Donor Recruitment Samples.** *PLoS One* 2016, **11:**e0165810.

3. Cascella R, Stocchi L, Strafella C, Mezzaroma I, Mannazzu M, Vullo V, Montella F, Parruti G, Borgiani P, Sangiuolo F, et al: **Comparative analysis between saliva and buccal swabs as source of DNA: lesson from HLA-B*57:01 testing.** *Pharmacogenomics* 2015, **16:**1039-1046.

4. Livy A, Lye S, Jagdish CK, Hanis N, Sharmila V, Ler LW, Pramod B: **Evaluation of Quality of DNA Extracted from Buccal Swabs for Microarray Based Genotyping.** *Indian Journal of Clinical Biochemistry* 2012, **27:**28-33.

5. Koni AC, Scott RA, Wang G, Bailey ME, Peplies J, Bammann K, Pitsiladis YP: **DNA yield and quality of saliva samples and suitability for large-scale epidemiological studies in children.** *Int J Obes (Lond)* 2011, **35 Suppl 1:**S113-118.
