# Supplementary material for: Evaluation of buccal swabs for pharmacogenetics
Source: BMC Res Notes. 2018 Jun 14;11:382. doi: 10.1186/s13104-018-3476-5 (PMC6000964; doi:10.1186/s13104-018-3476-5)
Supplement: Supplementary file 2 — Additional file 2. List of SNPs on the OpenArray panel. [file 13104_2018_3476_MOESM2_ESM.docx]

**Additional file 2** List of SNPs on the OpenArray panel

| **Assay ID** | **Gene** | **rsID** |
| --- | --- | --- |
| C_469857_10 | CYP2C19 | rs12248560 |
| C_1329192_10 | CYP2C9 | rs4918758 |
| C_2222771_40 | CYP2D6 | rs28371706 |
| C_2228708_20 | G6PD | rs5030868 |
| C_2451870_10 | HLA-B*58:01 tag | rs3117583 |
| C_7473918_10 | VKORC1 | rs7294 |
| C_8692805_10 | FLOT1 | rs3909184 |
| C_11195391_10 | HLA-A*31:01 tag | rs1633021 |
| C_11484460_40 | CYP2D6 | rs1065852 |
| C_11764545_20 | ADD1 | rs4961 |
| C_25625805_10 | CYP2C9 | rs1799853 |
| C_25986767_70 | CYP2C19 | rs4244285 |
| C_26544178_30 | HLA-B*15:02 tag | rs2844682 |
| C_27102414_10 | CYP2D6 | rs1135840 |
| C_27102425_10 | CYP2D6 | rs16947 |
| C_27102431_D0 | CYP2D6 | rs3892097 |
| C_27104892_10 | CYP2C9 | rs1057910 |
| C_27531918_10 | CYP2C19 | rs72552267 |
| C_27861809_10 | CYP2C19 | rs4986893 |
| C_27861810_10 | CYP2C19 | rs56337013 |
| C_29403047_10 | ACE | rs4341 |
| C_30403261_20 | VKORC1 | rs9923231 |
| C_30633906_10 | SLCO1B1 | rs4149056 |
| C_30634127_10 | CYP2C19 | rs72558186 |
| C_30634130_30 | CYP2C19 | rs41291556 |
| C_30634136_10 | CYP2C19 | rs28399504 |
| C_32407229_60 | CYP2D6 | rs5030656 |
| C_32407232_50 | CYP2D6 | rs35742686 |
| C_32407243_20 | CYP2D6 | rs5030655 |
| C_34816116_20 | CYP2D6 | rs28371725 |
