# Supplementary figures and images for: Evaluation of buccal swabs for pharmacogenetics
Source: BMC Res Notes. 2018 Jun 14;11:382. doi: 10.1186/s13104-018-3476-5 (PMC6000964; doi:10.1186/s13104-018-3476-5)

**Additional file 3** Copy number calls for DNA samples genotyped on a CYP2D6 CNV TaqMan Assay

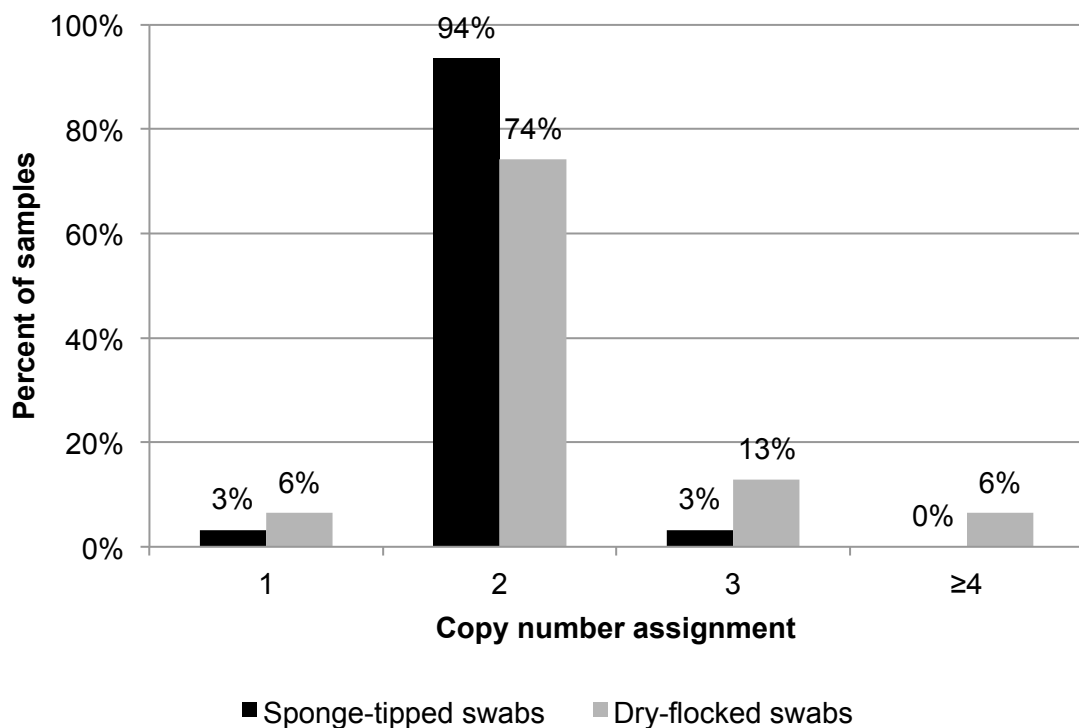

Supplement: Supplementary file 3 — Additional file 3. Copy number calls for DNA samples genotyped on a CYP2D6 CNV TaqMan Assay. [file 13104_2018_3476_MOESM3_ESM.pdf]
